# Tumor Markers in Pleural Fluid: A Comprehensive Study on Diagnostic Accuracy

**DOI:** 10.3390/diagnostics15020204

**Published:** 2025-01-17

**Authors:** Vladimir Aleksiev, Daniel Markov, Kristian Bechev

**Affiliations:** 1 Department of Thoracic Surgery, UMHAT “Kaspela”, 4001 Plovdiv, Bulgaria; 2 Department of Cardiovascular Surgery, Medical University of Plovdiv, 4002 Plovdiv, Bulgaria; 3 Department of Clinical Pathology, UMHAT “Pulmed”, 4002 Plovdiv, Bulgaria; 4 Department of General and Clinical Pathology, Medical University of Plovdiv, 4002 Plovdiv, Bulgaria; 5 Department of Neurosurgery, UMHAT “Pulmed”, 4002 Plovdiv, Bulgaria

**Keywords:** malignant pleural effusion, tumor marker, diagnosis

## Abstract

**Background/Objectives**: Malignant pleural effusions (MPEs) pose a significant challenge in clinical practice and exert a considerable socio-economic burden on the healthcare system, affecting approximately 1 million individuals annually. These effusions are a leading cause of debilitating dyspnea and a diminished quality of life among cancer patients, with distant metastasis to the pleural layers occurring in about 20% of cases during treatment. **Methods**: A cross-sectional, observational case-control study was conducted on 151 Bulgarian patients with a hydrothorax. The control group included 72 patients with benign diseases, confirmed via biopsy, with 38 having inflammatory and 34 non-inflammatory pleural effusions. The other 79 patients had malignant pleural involvement. These groups are representative of the main types of pleural pathology. **Results**: The study found that all of the tumor markers, except for PIVKA-II (Protein induced by vitamin K absence-II), showed statistically significant differences between the malignant and non-malignant patient groups, with CAE (carcinoembryonic antigen) and CA19-9 showing the most notable differences. The Receiver Operating Characteristic (ROC) analysis revealed that CA72-4 had the best ability to distinguish between the two groups, while PIVKA was the weakest, with optimal cut-off values for all of the relevant tumor markers being derived using the Youden index. **Conclusions**: In conclusion, our study highlights the transformative potential of pleural fluid tumor markers as precise and minimally invasive resources for distinguishing malignant from non-malignant pleural effusions. These findings pave the way for improved diagnostic accuracy and personalized clinical management, addressing a critical gap in the care of patients with pleural pathologies.

## 1. Introduction

The fact that approximately 40% of malignant pleural effusions do not yield positive cytology results during pleural puncture highlights the need for a panel of diagnostic methods to complement the available biochemical and cytological examinations [1]. A novel concept in the treatment of pleural effusions is the possibility to analyze pleural fluid for the presence of tumor markers [2].

Since pleural fluid is a filtrate of blood serum, its composition should reflect that of circulating blood [3]. Therefore, elevated levels of tumor markers in the circulation would also be expected to increase in the pleural fluid. Another theory suggests that pleural carcinomatosis leads to elevated tumor markers in pleural fluid, where they enter directly from metastases on the pleural lining [4]. Additionally, due to the impaired drainage of pleural fluid resulting from tumor invasion, the clearance of tumor markers is impaired, leading to elevated levels [5]. Potential possibilities for further development include creating an optimal panel of tumor markers based on the most common types of metastatic diseases affecting the pleura, which should be considered in conjunction with an extensive biochemical and cytological analysis of the pleural fluid. This approach will provide the most relevant data to help unravel the complexities of metastatic pleural effusions [6].

The assessment of tumor markers is a moderately expensive method that can become more cost-effective with a targeted search. The most studied tumor marker to date is carcinoembryonic antigen (CEA), which demonstrates a diagnostic sensitivity of 50–60%. An intriguing study conducted by Miedouge [7] demonstrated that the following tumor markers exhibit the corresponding percentages of diagnostic sensitivity:

- CEA: 60.0%;
- CA15-3: 63.7%;
- CYFRA: 42.8%;
- CA-19-9: 20.9%;
- CA72-4: 68.4%;
- SCC: 5.6%;
- NSE: 18.1%.

Some markers show a high sensitivity for certain diseases compared to others. Miedouge and his colleagues found that the panel of CEA, CA15-3, CYFRA, and NSE showed the most promising results, identifying 83.9% of cytologically negative non-lymphomatous malignant pleural effusions. The ability of tumor markers to predict the underlying histological type of neoplasia was examined using descriptive analysis. NSE, SCC, and CYFRA were shown to be the most reliable for differentiating adenocarcinomas, small cell lung carcinoma, squamous cell carcinoma, and mesothelioma. Their predictive accuracy reached 89.4% across all cases, with the highest accuracy observed for adenocarcinoma. However, identifying the different possible locations of adenocarcinoma proved challenging, as only 64.8% of adenocarcinomas were accurately grouped.

Despite their benefits, tumor markers demonstrate low diagnostic specificity when it comes to their quantitative assessment in pleural fluid [8]. For malignant pleural effusions, the listed tumor markers exhibit similar diagnostic capabilities. The sensitivity of pleural CEA, according to Miedouge’s findings, aligns with results from known studies in the literature, confirming its significance in diagnosing carcinomas. CA72-4 has confirmed its relevance in detecting metastatic adenocarcinoma and squamous cell carcinoma. CA15-3 is expressed in pleural fluid in cases of carcinomatosis resulting from invasive ductal adenocarcinoma of the breast and adenocarcinomas of other origins. CYFRA shows good predictive results in diagnosing pleural mesothelioma. Importantly, pleural fluid serves as a suitable medium for analyzing tumor markers released by cancer cells. In several patients, their levels were found to be significantly elevated, even exceeding concentrations in serum.

Numerous studies have established the use of discriminant statistical analysis and logistic regression analysis to highlight the advantages of the quantitative assessment of tumor markers in pleural fluid. Serum concentrations of CYFRA 21-1 and NSE, as well as pleural NSE, have been shown to be adequate predictors of pleural malignancy. Their combined application has established statistical significance [9]. According to a notable study conducted by Volaric and his colleagues involving a sample of 73 men and 27 women, the average age of the patients was 71 years, with 88 diagnosed with unilateral pleural effusion. Based on Light’s criteria, a pleural exudate was diagnosed in 71% of cases, while the remaining met the criteria for transudate.

The gender distribution revealed that 39 men and 16 women were in the malignant group, while 34 men and 11 women were in the non-malignant group of pleural effusions. The analysis showed that the concentrations of tumor markers CEA, NSE, CA-125, and CYFRA 21-1 were significantly elevated in malignant pleural effusions.

Many of the studies available in the literature, investigating the diagnostic significance of tumor markers in pleural fluid, employ the capabilities of retrospective analysis and have been conducted in standalone hospital centers without a validated cohort group. This is why Zhai and his collaborators [10] conducted the first derivative and validation study in China, examining the diagnostic efficacy of CEA, CA125, CA15-3, and CA19-9. Their results further reinforce the notion that tumor markers are useful for differentiating benign from malignant pleural effusions, showing dramatically elevated levels in the latter group. Among the examined markers, CEA demonstrated the highest diagnostic accuracy, consistent with existing information. It showed an 84.7% sensitivity and a 90.9% specificity at a laboratory threshold of 2.42 ng/mL. Its elevated levels are associated with direct pleural infiltration. CA15-3 was reported to have a diagnostic specificity of 97.6% but a dramatically low sensitivity of 63.1%. Therefore, the standalone use of this tumor marker is not recommended. The results regarding CA125 and CA19-9 were contradictory. For example, CA125 had a laboratory threshold ranging from 558.7 to 383.9 U/mL, while CA19-9 ranged from 9.3 to 11.1 U/mL. These discrepancies were attributed to tumor heterogeneity and varying stages of metastatic disease. This leads to the conclusion that perhaps these two markers are not the most suitable for investigating malignant pleural effusions.

To confirm a diagnosis of malignant pleural effusion, we should use the tumor marker with the highest sensitivity, while, to rule out malignancy, the tumor marker should have a proven high specificity [11,12,13,14,15,16]. A given marker may excel at confirming a diagnosis but not at excluding it, or vice versa. To further enhance the diagnostic sensitivity and specificity, many authors recommend the combined examination of tumor markers in pleural fluid and serum, as well as calculating their pleural gradient [17,18,19,20,21,22].

The conflicting opinions regarding the use of tumor markers for diagnosing malignant pleural effusions unanimously agree on one point: the quantitative determination of tumor markers in pleural fluid could be indicated for patients with an undetermined cause of hydrothorax, in the presence of a high clinical suspicion of malignancy despite inconclusive cytological analysis, but with a known oncological history [23,24,25].

## 2. Materials and Methods

In order to achieve the set objectives, a cross-sectional, observational case-control study was conducted on a Bulgarian population of patients with hydrothorax.

A total of 151 patients participated in the analysis. In the control group of 72 patients, a benign disease was diagnosed and confirmed through subsequent biopsy. Of these, 38 cases were identified as inflammatory, while 34 were verified as pleural effusions of non-inflammatory origin. Malignant pleural involvement was confirmed in 79 patients. These two groups are representative of the main types of pleural pathology.

Pleural fluid was obtained using a closed container for biological material. The biological material was collected during thoracentesis or intraoperatively during VATS. A portion of the collected pleural fluid was used to determine biochemical parameters, while the remaining fluid was set aside for the analysis of tumor markers and cytological examination. All of the analyses were performed using the clinico-chemical analyzer “Beckman Coulter”, model AU480, according to the original programs.

The choice of statistical methods was made according to the objectives of the study, the type of variables, and the established practices in the scientific research in the field of thoracic surgery. The systematization, processing, and analysis of the primary data in the form of quantitative and qualitative variables were carried out using the statistical software package IBM SPSS Statistics 27.0.1. The analysis and conclusions from the study were drawn after a summarized presentation of the empirical results in tabular form and were illustrated with the corresponding graphs. The graphical analysis was performed using MS Office 365. To objectify the results of the analyses conducted, the following statistical-mathematical methods were used:

Mann–Whitney Wilcoxon Test: A non-parametric statistical analysis used to compare two independent groups. Its purpose is to determine whether the distribution of the two populations differs significantly from each other.

Kolmogorov–Smirnov’s One-Sample Test: A non-parametric test used to check whether a given sample follows a specific distribution. It compares the empirical distribution of data with the theoretical distribution.

Independent Samples *t*-test: A parametric test used to compare the means of two independent groups.

Levene’s Test for Equality of Variances: A statistical test used to check for equality of variances among two or more groups.

Correlation Analysis: A statistical method used to assess the relationship between two or more variables. It helps to understand whether changes in one independent variable are associated with changes in another dependent variable. It does not establish a causal relationship but measures the degree of association between the variables.

Receiver Operating Characteristic (ROC) Curve: A graphical method used to evaluate the performance of binary classifiers. It demonstrates the behavior of the classifier at different threshold values for decision making.

Youden Index: A statistical measure used to evaluate diagnostic tests. It combines sensitivity and specificity, allowing for a comprehensive assessment of the test’s performance in distinguishing between positive and negative cases.

To reject the null hypothesis, we looked for values of *p* ≤ 0.05 in all of the analyses. When it was rejected and *p* ≤ 0.05, the alternative hypothesis (H1) could be accepted, which means that there was a statistically significant difference or relationship.

## 3. Results

To demonstrate the significance of the examined tumor markers in pleural effusions, we used the previously mentioned methods to assess the normal distribution of the data. Applying the Kolmogorov–Smirnov test, we observed that all of the variables did not follow a normal distribution. The greatest deviation in the distribution was observed for CA19-9 (*p* = 0.453) and PIVKA (*p* = 0.447). The results of the analysis are presented in Table 1.

When we applied the Independent Samples *t*-test, which checked for the differences in the mean values between the two groups, and Levene’s test, which examined whether the two groups demonstrated equality of variances, we obtained the results shown in Table 2.

Of all the variables, only the values of PIVKA did not show a difference between the malignant and non-malignant groups. All of the other variables had a significant differences in mean values, with CAE and CA19-9 showing the most pronounced differences.

When we applied the Mann–Whitney U test and Wilcoxon W test, which again assessed the significance of the examined parameters, we saw that all of the tumor markers, except for PIVKA, demonstrated a statistically significant difference between the two groups, with *p* < 0.005. Therefore, these markers can be associated with the malignant nature of the effusion. The results are presented in Table 3.

Looking for the interrelationships among the examined tumor markers, we analyzed the correlation dependencies between them. The results of the correlation analysis are presented in Table 4, with the corresponding correlations marked for ease of reference.

To analyze the relationship between the sensitivity and specificity of tumor markers, and to derive example cut-off values, we decided to use the Receiver Operating Characteristic (ROC) curve for each of them.

**Figure 1:**
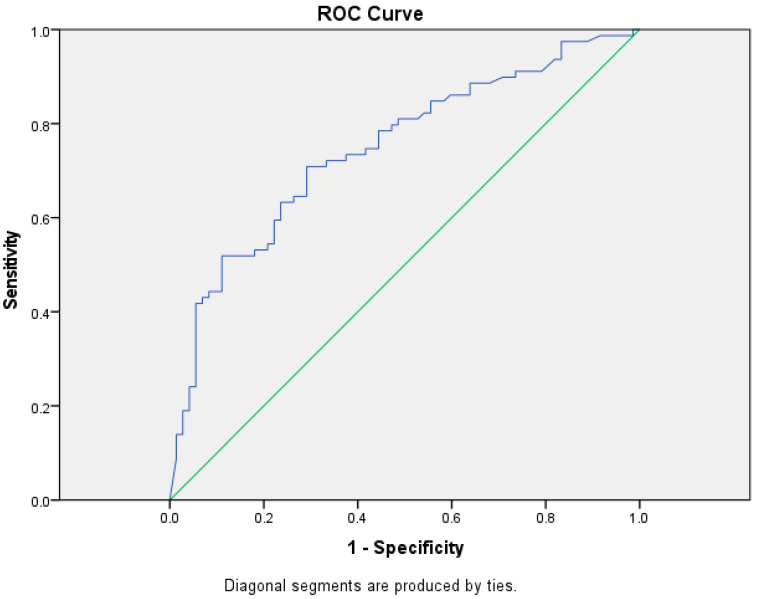
Receiver Operating Characteristic (ROC) curve for carcinoembryonic antigen (CEA), showing diagnostic sensitivity and specificity for malignant pleural effusions.

The curve consists of two axes. The X-axis illustrates the specificity (false-positive rate) and shows the proportion of false-positive cases. The Y-axis represents the sensitivity (true-positive rate) and indicates the proportion of correctly classified positive cases relative to the total number of patients. The green diagonal represents the line of random guessing, where the model has no predictive power (*p* = 0.5). In the exhibited table, the blue curve significantly exceeds the green one, confirming the hypothesis that the model has good classification abilities. The presented Table 5 is a visual analysis of the data obtained in Figure 1. It shows the results of the analysis of the Area Under the Curve (AUC) for the CEA marker. It is notable that the model demonstrates good discriminative ability (0.746) in classifying the two groups. This value indicates that this tumor marker has a 74.6% probability of correctly classifying a pleural effusion as malignant. Additionally, the small value of the standard error and the significant *p*-value confirm that the results are statistically significant.

**Figure 2:**
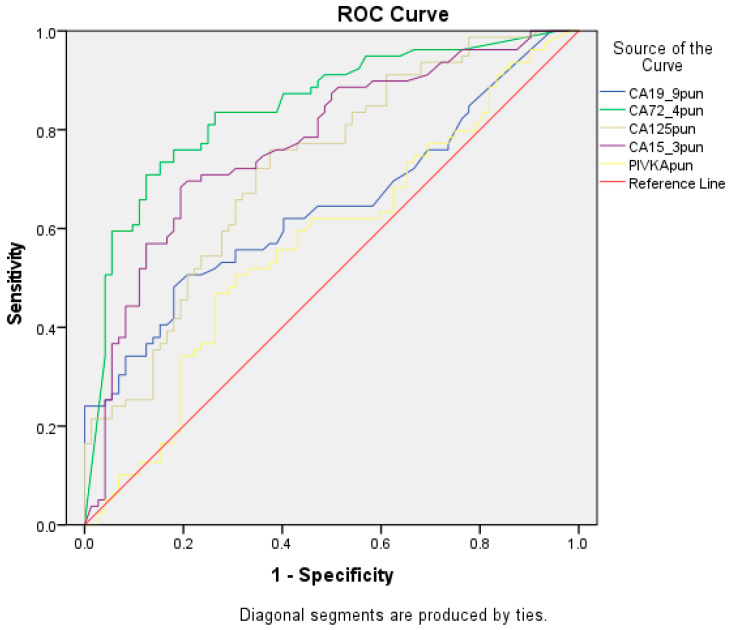
Comparative ROC curves for the CA19-9, CA72-4, CA125, and CA15-3 tumor markers, highlighting the diagnostic accuracy for the pleural effusion analysis.

We conducted an identical analysis for each of the examined tumor markers. The ROC curves are illustrated in Figure 2. The obtained results are presented in Table 6.

Based on the obtained AUC values, it is possible to predict the discriminative ability of each of the examined tumor markers. Thus, their ability to distinguish between malignant and non-malignant pleural effusions can be classified into the following four groups: very good (CA72-4), good (CA125; CA15-3), moderate (CA19-9), and weak (PIVKA). As can be seen, CA72-4 shows the best discriminative ability between the two groups, while PIVKA is classified as a statistically insignificant biomarker.

To differentiate positive from negative results in a diagnostic test, it is necessary to derive a cut-off value for the examined parameter. A cut-off or threshold value is a value that effectively separates the positive from the negative results.

Using the obtained ROC curves, we can identify the values at which the sensitivity and specificity are optimally balanced. For this purpose, we used the Youden index, which is calculated using the following formula:

J = Sensitivity + (1 − Specificity) − 1J = Sensitivity + (1 − Specificity) − 1J = Sensitivity + (1 − Specificity) − 1

Thus, for example, we obtained the following values presented in Table 7, which offer the best compromise between specificity and sensitivity.

According to our study, the tumor markers demonstrate the following performance characteristics:

- CEA with a cut-off of 1.8 shows a sensitivity of 70.9% and a specificity of 70.8%;
- CA19-9 with a cut-off of 3.75 shows a sensitivity of 48.1% and a specificity of 81.9%;
- CA72-4 with a cut-off of 4.52 shows a sensitivity of 70.9% and a specificity of 87.5%;
- CA125 with a cut-off of 544.43 shows a sensitivity of 75.9% and a specificity of 62.5%;
- CA15-3 with a cut-off of 9.65 shows a sensitivity of 68.4% and a specificity of 80.6%.

## 4. Discussion

The non-invasive nature of tumor marker analysis in pleural fluid offers a less burdensome alternative to more invasive procedures, particularly for frail patients or those with advanced disease. This aligns with modern approaches aimed at improving patient comfort while maintaining diagnostic rigor. The initial subtype classification of pleural effusion as transudative or exudative, using the traditional criteria proposed by Light, is relatively accurate, providing a correct diagnosis in nearly 100 percent of exudative pleural effusions [26]. However, a portion of transudative pleural effusions is incorrectly classified as exudative according to these criteria. This leads to a series of unnecessary investigations that could be avoided.

The significance of tumor markers for malignant pleural diseases lies in their ability to rule out or confirm the presence of malignancy. There is certainly no shortage of studies examining the diagnostic capabilities of tumor markers, but none present definitive results [27]. We can be certain that markers like CEA, CA15-3, and CYFRA 21-1 are elevated in malignant pleural effusions [28]. To demonstrate this, we applied the Mann–Whitney U test, which showed that all of the tumor markers we examined, except for PIVKA, are statistically significant between the two groups of patients.

There are few studies investigating the role of tumor markers in identifying malignant pleural effusions. In a study conducted by Porcel [29], 1575 patients with non-purulent pleural effusions were observed. Among them, 549 had confirmed pleural carcinomatosis, 284 had highly suspicious pleural carcinomatosis, but it was not proven, and 742 had benign pleural effusions. Elevated levels of the tumor markers CEA and CA15-3 were found in the pleural fluid in the malignant group. A proposed cut-off value for CEA was >45 ng/mL, which was exceeded in 30% of cases. The cut-off value for CA15-3 was >77 UI/L, which was exceeded in 19% of patients. Both tumor markers were elevated in 41% of the patients. When applying the ROC analysis, an Area Under the Curve (AUC) of 0.819 (95% CI: 0.793–0.845) was obtained for CEA, and 0.822 (95% CI: 0.796–0.847) for CA15-3. Additionally, it was shown that the combination of tumor markers and the cytopathological examination of pleural fluid diagnosed malignant pleural effusions in 14% more cases.

Similar results were demonstrated by Khalaf in his study [30], where he examined the values of CEA, CA15-3, CYFRA21-1, CA19-9, and CA125 in 281 patients with malignant and benign pleural effusions. It was established that all of the tumor markers were significantly elevated in the malignant group. The Areas Under the Curve yielded the following results: CEA (0.81), CA15-3 (0.78), CYFRA21-1 (0.75), CA19-9 (0.65), and CA125 (0.65). The best results regarding the sensitivity were obtained with the combined use of CEA and CA-15-3 (94%). Similar observations were noted by Antonangelo [26].

Other studies validating the role of tumor markers in the diagnostic panel for malignant pleural effusions include the A Study Investigating Markers in Pleural Effusion (SIMPLE) [5], which is a prospective, double-blind study with a significant patient sample lasting 3 years, and the Diagnostic and Prognostic Biomarkers in the Rational Assessment of Mesothelioma (DIAPHRAGM) [31].

In his work, Trape examined 402 patients, 122 of whom had malignant pleural effusions. Threshold values for each tumor marker were derived with a combined sensitivity of 63.9%. The proposed values were 60 μg/L for CEA, 80 KU/L for CA15-3, 209 KU/L for CA19-9, and 21 KU/L for CA72-4. Other studies proposed varying values of 40 to 50 μg/L for CEA, 53 to 75 KU/L for CA15-3, and 8.9 to 16 KU/L for CA72-4 [32,33].

According to our analyses, the tumor marker CEA shows very good performance, with an AUC = 0.746. Furthermore, it is statistically significant between the malignant and benign groups, as *p* < 0.001. Its confidence interval is also relatively narrow, making it reliable for differentiating between our two groups.

The tumor marker CA19-9 shows a moderate diagnostic accuracy, with an AUC = 0.644. Additionally, since *p* = 0.002, it can be said that the marker is reliable, and the results are statistically significant.

Based on the sample, CA72-4 shows a high diagnostic accuracy, with an AUC = 0.845, making it an excellent indicator of malignancy. This marker demonstrates the best result of all examined, and is statistically significant with *p* < 0.001. Some authors even proposed this marker as a leading method for differentiating malignant from benign pleural effusions [34].

Regarding the results for CA15-3, we can say that it has a good diagnostic accuracy, with an AUC = 0.773, and the results are statistically significant, with *p* < 0.001.

Only PIVKA demonstrates a low diagnostic value, with an AUC = 0.571, indicating that this tumor marker is a weak indicator for differentiating malignant from benign pleural effusions. Furthermore, the results are not statistically significant, as *p* = 0.132.

The study of tumor markers in pleural fluid may offer significant advantages in terms of cost and method efficiency. Although these markers show a high specificity, their relatively low sensitivity limits their application. Nevertheless, the combined use of tumor markers and traditional diagnostic methods, such as Light’s criteria and cytological analysis, could be incorporated into new diagnostic algorithms. These would leverage the strengths of individual tests to provide comprehensive, evidence-based pathways for managing pleural effusions. There is disagreement regarding which values should be accepted as thresholds when examining tumor markers in pleural fluid. In a study by Zhang, a sample of 619 individuals diagnosed with pleural effusions over two years was observed. Among them, 274 patients had lung cancer, and 74 were diagnosed with benign pleural effusions. A sensitivity of 89.8% and specificity of 98.6% for CEA (AUC of 0.978) was found at a threshold value of 5.23 ng/mL. According to data from the study, CYFRA 21-1 demonstrated a sensitivity of 67.9% with a specificity of 90.5% (AUC of 0.853) at a threshold value of 31.39 ng/mL.

According to another study, elevated levels of CA125, CEA, CA15-4, CA19-9, CYFRA21-1, and NSE were found, indicating that the greatest diagnostic value lies with CEA at a threshold value of 3.7 ng/mL (AUC [0.890 (0.871–0.907)]). This is further strengthened when CEA levels are examined in conjunction with CYFRA21-1 levels, as the sensitivity of the combined examination reaches 79.9%, and the specificity is 95.7%.

Other studies recommend the combined examination of CEA and CA15-3, as well as CEA and CA19-9, proving that the levels of these tumor markers are significantly elevated in malignant pleural effusions, but remain with a low sensitivity [35].

Feng [36] conducted a study comparing 81 pleural effusions associated with lung adenocarcinoma and 96 benign pleural effusions. The levels of CEA, CYFRA21-1, and CA19-9 were examined. According to the analyses, the optimal threshold values were determined as 0.93, 0.85, and 0.81, respectively. The combined examination of these three markers increased the sensitivity to 95.06%, with an AUC = 0.95.

The importance of the combined examination of tumor markers in pleural fluid is also evident in Yang’s analysis. A series of bibliographic databases were reviewed, and among 20 studies investigating the levels of tumor markers in pleural fluid, the following sensitivity/specificity values were derived: CEA + CA 125, 0.65/0.98; CEA + CA 15-3, 0.64/0.98; CEA + CA 19-9, 0.58/0.98; CEA + CYFRA 21-1, 0.82/0.92; CA 15-3 + CYFRA 21-1, 0.88/0.94. In conclusion, it is suggested that, for unexplained pleural effusions, the combined examination of CA15-3, CEA, and CA19-9 is recommended.

According to the analyses we conducted, we can calculate the sensitivity and specificity of the thresholds we derived as follows: CEA (75.9%/55.6%), CA19-9 (48.1%/18.1%), CA72-4 (70.9%/12.5%), CA125 (75.9%/37.5%), and CA15-3 (68.4%/80.6%).

Sensitivity measures the ability of the test to detect disease when it is present. It represents the percentage of people who have malignant pleural effusion and whose positivity for the tumor marker will correctly diagnose the condition. Specificity measures the test’s ability to identify patients who do not have malignant pleural effusions. It measures the percentage of people without carcinomatosis who are correctly classified as benign.

The results we obtained largely reflect global data. Of course, the thresholds derived are not definitive and can only guide us in extreme deviations beyond the reference parameters. Current guidelines for the management of pleural effusions emphasize cytological and biochemical analyses as first-line diagnostic tools [37]. However, the demonstrated diagnostic value of markers such as CEA, CA15-3, and CA72-4 calls for their inclusion as supplementary tools, particularly in cases with a high clinical suspicion of malignancy but non-diagnostic initial results. The results of this study could contribute to the development of updated clinical recommendations, including thresholds and decision trees incorporating tumor marker analysis.

Despite these advantages, the relatively low sensitivity of individual markers highlights the importance of combining them for enhanced diagnostic accuracy. Further research is needed to validate these findings across diverse populations and healthcare settings. Additionally, the standardization of laboratory thresholds and techniques will be essential to facilitate broader clinical adoption.

## 5. Conclusions

In conclusion, diagnosing malignant diseases of the pleura presents a diagnostic challenge, even for experienced clinicians. Their frequency and diversity concerning the etiology and pathogenic mechanisms of the disease demonstrate the need for a multidisciplinary and patient-centered approach. Each hydrothorax should be considered and treated individually, which provides new perspectives for optimizing its diagnosis and treatment.

The established approaches to differentiate malignant from benign pleural effusions must be reassessed, especially in the era of modern laboratory and diagnostic methods. Undoubtedly, the patterns described by Light have remained valid nearly 60 years after his initial publications. However, in line with contemporary efforts to improve the quality of life for cancer patients, the introduction of innovative markers and techniques opens new horizons for enhancing the diagnosis of pleural neoplasms.

By determining the diagnostic significance of tumor markers in malignant pleural effusions, we can conclude their undeniable advantages regarding minimal invasiveness and justification. It is clear that some tumor markers hold a significantly greater diagnostic value than others. Despite the available results and conducted studies, the need for additional data remains.

The current progress in science and medicine places us on the brink of new possibilities in diagnosing malignant diseases of the pleura. We believe that, by combining traditional experience with innovative approaches, we can achieve significant advances in improving outcomes and the quality of life for patients. However, this progress will depend on ongoing research and the medical community’s efforts to integrate new technologies into our clinical practice.

## Figures and Tables

**Table 1:** Kolmogorov–Smirnov test results indicating normality distribution for tumor markers.

One-Sample Kolmogorov–Smirnov Test							
		CEApun	CA19_9pun	CA72_4pun	CA125pun	CA15_3pun	PIVKApun
N		151.00	151.00	151.00	151.00	151.00	151.00
Normal Parameters	Mean	109.36	138.61	120.49	1303.59	77.59	210.04
	Std. Deviation	266.88	461.52	201.02	1477.59	189.80	852.71
Most Extreme Differences	Absolute	0.37	0.45	0.37	0.20	0.35	0.45
	Positive	0.37	0.45	0.37	0.20	0.35	0.45
	Negative	−0.34	−0.38	−0.27	−0.19	−0.34	−0.40
Test Statistic		0.37	0.45	0.37	0.20	0.35	0.45
Asymp. Sig. (2-tailed)		0.000	0.000	0.000	0.000	0.000	0.000

**Table 2:** The *t*-test applied for the values of all tumor markers.

Independent Samples Test										
		Levene’s Test for Equality of Variances		*t*-Test for Equality of Means						
		F	Sig.	t	df	Sig. (2-Tailed)	Mean Difference	Std. Error Difference	95% Confidence Interval of the Difference	
									Lower	Upper
CEApun	Equal variances assumed	38.84	0.00	−3.57	149.00	0.00	−149.71	41.87	−232.45	−66.98
	Equal variances not assumed			−3.69	108.60	0.00	−149.71	40.55	−230.11	−69.32
CA19_9pun	Equal variances assumed	50.13	0.00	−3.50	149	0.00	−253.98	72.52	−397.29	−110.67
	Equal variances not assumed			−3.66	78.08	0.00	−253.98	69.21	−391.78	−116.18
CA72_4pun	Equal variances assumed	162.42	0.00	−6.39	149.00	0.00	−186.00	29.12	−243.54	−128.47
	Equal variances not assumed			−6.59	109.34	0.00	−186.00	28.21	−241.92	−130.08
CA125pun	Equal variances assumed	22.50	0.00	−4.45	149.00	0.00	−1009.40	226.96	−1457.88	−560.93
	Equal variances not assumed			−4.56	125.23	0.00	−1009.40	221.55	−1447.87	−570.94
CA15_3pun	Equal variances assumed	8.44	0.00	−2.39	149.00	0.02	−72.90	30.45	−133.06	−12.73
	Equal variances not assumed			−2.43	142.87	0.02	−72.90	30.03	−132.26	−13.54
PIVKApun	Equal variances assumed	1.46	0.23	0.56	149.00	0.58	77.90	139.25	−197.27	353.07
	Equal variances not assumed			0.55	117.90	0.58	77.90	142.07	−203.44	359.24

**Table 3:** The Mann–Whitney U Test applied for our tumor markers.

Test Statistics						
	CEApun	CA19_9pun	CA72_4pun	CA125pun	CA15_3pun	PIVKApun
Mann–Whitney U	1443.50	2023.00	879.50	1574.50	1291.50	2440.00
Wilcoxon W	4071.50	4651.00	3507.50	4202.50	3919.50	5068.00
Z	−5.22	−3.07	−7.35	−4.73	−5.78	−1.51
Asymp. Sig. (2-tailed)	0.00	0.00	0.00	0.00	0.00	0.13

**Table 4:** Correlation analysis of tumor marker levels, highlighting significant interrelationships.

Correlations							
		CEApun	CA19_9pun	CA72_4pun	CA125pun	CA15_3pun	PIVKApun
CEA	Pearson Correlation	1.00	0.42	0.33	0.37	0.12	−0.08
	Sig. (1-tailed)		0.00	0.00	0.00	0.07	0.16
	N	151.00	151.00	151.00	151.00	151.00	151.00
CA19-9	Pearson Correlation	0.42	1.00	0.23	0.45	−0.03	−0.05
	Sig. (1-tailed)	0.00		0.00	0.00	0.34	0.26
	N	151.00	151.00	151.00	151.00	151.00	151.00
CA72-4	Pearson Correlation	0.33	0.23	1.00	0.36	0.35	−0.11
	Sig. (1-tailed)	0.00	0.00		0.00	0.00	0.09
	N	151.00	151.00	151.00	151.00	151.00	151.00
CA125	Pearson Correlation	0.37	0.45	0.36	1.00	0.05	−0.09
	Sig. (1-tailed)	0.00	0.00	0.00		0.26	0.15
	N	151.00	151.00	151.00	151.00	151.00	151.00
CA15-3	Pearson Correlation	0.12	−0.03	0.35	0.05	1.00	−0.08
	Sig. (1-tailed)	0.07	0.34	0.00	0.26		0.17
	N	151.00	151.00	151.00	151.00	151.00	151.00
PIVKA	Pearson Correlation	−0.08	−0.05	−0.11	−0.09	−0.08	1.00
	Sig. (1-tailed)	0.16	0.26	0.09	0.15	0.17	
	N	151.00	151.00	151.00	151.00	151.00	151.00

**Table 5:** The area under the curve for CEA.

Area Under the Curve				
Area	Std. Error	Asymptotic Sig.	Confidence Interval	
			Lower Bound	Upper Bound
0.75	0.04	0.00	0.67	0.82

**Table 6:** Areas Under the Curve for said tumor markers.

Area Under the Curve					
Test Result Variable(s)	Area	Std. Error	Asymptotic Sig.	Confidence Interval	
				Lower Bound	Upper Bound
CA19_9pun	0.64	0.04	0.00	0.56	0.73
CA72_4pun	0.85	0.03	0.00	0.78	0.91
CA125pun	0.72	0.04	0.00	0.64	0.80
CA15_3pun	0.77	0.04	0.00	0.70	0.85
PIVKApun	0.57	0.05	0.13	0.48	0.66

**Table 7:** Optimal cut-off values and corresponding sensitivity and specificity for tumor markers using Youden’s index.

Tumor Marker	Cut-Off Value	Youden Index
CEA	>1.8	0.417
CA19-9	>3.75	0.3
CA72-4	>4.52	0.584
CA125	>544.43	0.382
CA15-3	>9.65	0.489

## Data Availability

Data supporting the reported results cannot be disclosed due to institutional privacy and ethical restrictions.

## References

[1] Kaul V., McCracken D.J., Rahman N.M., Epelbaum O. (2019). Contemporary Approach to the Diagnosis of Malignant Pleural Effusion. Ann. Am. Thorac. Soc..

[2] Skok K., Hladnik G., Grm A., Crnjac A. (2019). Malignant Pleural Effusion and Its Current Management: A Review. Medicina.

[3] Clive A.O., Kahan B.C., Hooper C.E., Bhatnagar R., Morley A.J., Zahan-Evans N., Bintcliffe O.J., Boshuizen R.C., Fysh E.T.H., Tobin C.L. (2014). Predicting survival in malignant pleural effusion: Development and validation of the LENT prognostic score. Thorax.

[4] Ermin S., Özdogan Y., Batum Ö., Yilmaz U. (2022). The role of LENT and PROMISE scores in predicting survival in malignant pleural effusion. Lung India.

[5] Duffy M.J., Bonfrer J.M., Kulpa J., Rustin G.J., Soletormos G., Torre G.C., Tuxen M.K., Zwirner M. (2005). CA125 in ovarian cancer: European Group on Tumor Markers (EGTM) guidelines for clinical use. Int. J. Gynecol. Cancer.

[6] Harris L., Fritsche H., Mennel R., Norton L., Ravdin P., Taube S., Somerfield M.R., Hayes D.F., Bast R.C. (2007). American Society of Clinical Oncology 2007 update of recommendations for the use of tumor markers in breast cancer. J. Clin. Oncol..

[7] Miédougé M., Rouzaud P., Salama G., Pujazon M.C., Vincent C., Mauduyt M.A., Reyre J., Carles P., Serre G. (1999). Evaluation of seven tumour markers in pleural fluid for the diagnosis of malignant effusions. Br. J. Cancer.

[8] Duffy M.J., O’Donovan N., Crown J. (2011). Use of molecular markers for predicting therapy response in cancer patients. Cancer Treat. Rev..

[9] Volarić D., Flego V., Žauhar G., Bulat-Kardum L. (2018). Diagnostic value of tumour markers in pleural effusions. Biochem. Med..

[10] Zhai K., Wang W., Wang Y., Liu J.Y., Zhou Q., Shi H.Z. (2017). Diagnostic accuracy of tumor markers for malignant pleural effusion: A derivation and validation study. J. Thorac. Dis..

[11] Awadallah S.F., Bowling M.R., Sharma N., Mohan A. (2019). Malignant pleural effusion and cancer of unknown primary site: A review of literature. Ann. Transl. Med..

[12] Duffy M.J. (2007). Role of tumor markers in patients with solid tumors: A critical review. Eur. J. Int. Med..

[13] Rustin G.J., van der Burg M.E., Griffin C.L., Guthrie D., Lamont A., Jayson G.C., Kristensen G., Mediola C., Coens C., Qian W. (2010). MRC OV05; EORTC 55955 investigator: Early versus delayed treatment of relapsed ovarian cancer (MRC OV05/EORTC 55955): A randomised trial. Lancet.

[14] Hernandez J., Thompson I.M. (2004). Prostate-specific antigen: A review of the validation of the most commonly used cancer biomarker. Cancer.

[15] Molina R., Barak V., van Dalen A., Duffy M.J., Einarsson R., Gion M., Goike H., Lamerz R., Nap M., Sölétormos G. (2005). Tumor markers in breast cancer—European Group on Tumor Markers recommendations. Tumour Biol..

[16] Sturgeon C.M., Duffy M.J., Stenman U.H., Lilja H., Brünner N., Chan D.W., Babaian R., Bast R.C., Dowell B., Esteva F.J. (2008). National Academy of Clinical Biochemistry laboratory medicine practice guidelines for use of tumor markers in testicular, prostate, colorectal, breast and ovarian cancers. Clin. Chem..

[17] Lin D., Shen L., Luo M., Zhang K., Li J., Yang Q., Zhu F., Zhou D., Zheng S., Chen Y. (2021). Circulating tumor cells: Biology and clinical significance. Signal Transduct. Target. Ther..

[18] Sai B., Xiang J. (2018). Disseminated tumour cells in bone marrow are the source of cancer relapse after therapy. J. Cell. Mol. Med..

[19] Borriello L., Coste A., Traub B., Sharma V.P., Karagiannis G.S., Lin Y., Wang Y., Ye X., Duran C.L., Chen X. (2022). Primary tumor associated macrophages activate programs of invasion and dormancy in disseminating tumor cells. Nat. Commun..

[20] Wang W.-C., Zhang X.-F., Peng J., Li X.-F., Wang A.-L., Bie Y.-Q., Shi L.-H., Lin M.-B., Zhang X.-F. (2018). Survival Mechanisms and Influence Factors of Circulating Tumor Cells. BioMed Res. Int..

[21] Rastel D., Ramaioli A., Cornillie F., Thirion B. (1994). CYFRA 21-1, a sensitive and specific new tumour marker for squamous cell lung cancer. Report of the first European multicentre evaluation. Eur. J. Cancer.

[22] Isgrò M.A., Bottoni P., Scatena R. (2015). Neuron-Specific Enolase as a Biomarker: Biochemical and Clinical Aspects. Adv. Exp. Med. Biol..

[23] Koch T., Eiffert H., Spindler M.B. (1989). Die Relevanz des neuen Tumormarkers SCC (Squamous Cell Carcinoma Antigen) für die Diagnostik und Verlaufskontrolle von Plattenepithelkarzinomen im Kopf-Hals-Bereich. HNO.

[24] Zhu H. (2022). Squamous Cell Carcinoma Antigen: Clinical Application and Research Status. Diagnostics.

[25] Betz D., Fane K. (2024). Human Chorionic Gonadotropin. StatPearls [Internet].

[26] Antonangelo L., Sales R.K., Corá A.P., Acencio M.M., Teixeira L.R., Vargas F.S. (2015). Pleural fluid tumour markers in malignant pleural effusion with inconclusive cytologic results. Curr. Oncol..

[27] Villena V., López-Encuentra A., Echave-Sustaeta J., Martín-Escribano P., Ortuño-de-Solo B., Estenoz-Alfaro J. (2003). Diagnostic value of CA 549 in pleural fluid. Comparison with CEA, CA 15.3 and CA 72.4. Lung Cancer.

[28] Foresti V., Parisio E., De Filippi G. (1992). False positivity of tumor markers in pleural fluid of traumatic hemothorax. Recenti Prog. Med..

[29] Porcel J.M., Civit C., Esquerda A., Salud A., Bielsa S. (2017). Utilidad de la medición de CEA y CA 15-3 en los exudados pleurales no purulentos para diagnosticar malignidad: Experiencia de un único centro. Arch. Bronconeumol..

[30] Fazli Khalaf F., Asadi Gharabaghi M., Balibegloo M., Davari H., Afshar S., Jahanbin B. (2023). Pleural CEA, CA-15-3, CYFRA 21-1, CA-19-9, CA-125 discriminating malignant from benign pleural effusions: Diagnostic cancer biomarkers. Int. J. Biol. Markers.

[31] Tsim S., Kelly C., Alexander L., McCormick C., Thomson F., Woodward R., Foster J.E., Stobo D.B., Paul J., Maskell N.A. (2016). Diagnostic and Prognostic Biomarkers in the Rational Assessment of Mesothelioma (DIAPHRAGM) study: Protocol of a prospective, multicentre, observational study. BMJ Open.

[32] Han Y.Q., Yan L., Li P., Zhang L., Ouyang P.H., Hu Z.D. (2019). A Study Investigating Markers in PLeural Effusion (SIMPLE): A prospective and double-blind diagnostic study. BMJ Open.

[33] Ferrer J., Villarino M.A., Encabo G., Felip E., Bermejo B., Vilà S., Orriols R. (1999). Diagnostic utility of CYFRA 21-1, carcinoembryonic antigen, CA 125, neuron specific enolase, and squamous cell antigen level determinations in the serum and pleural fluid of patients with pleural effusions. Cancer.

[34] Villena V., López-Encuentra A., Echave-Sustaeta J., Martín-Escribano P., Ortuño-de-Solo B., Estenoz-Alfaro J. (1996). Diagnostic value of CA 72-4, carcinoembryonic antigen, CA 15-3, and CA 19-9 assay in pleural fluid: A study of 207 patients. Cancer.

[35] Yang Y., Liu Y.-L., Shi H.-Z. (2017). Diagnostic Accuracy of Combinations of Tumor Markers for Malignant Pleural Effusion: An Updated Meta-Analysis. Respiration.

[36] Feng M., Zhu J., Liang L., Zeng N., Wu Y., Wan C., Shen Y., Wen F. (2017). Diagnostic value of tumor markers for lung adenocarcinoma-associated malignant pleural effusion: A validation study and meta-analysis. Int. J. Clin. Oncol..

[37] Roberts M.E., Rahman N.M., Maskell N.A., Bibby A.C., Blyth K.G., Corcoran J.P., Edey A., Evison M., de Fonseka D., Hallifax R. (2023). British Thoracic Society Guideline for Pleural Disease. Thorax.

